# Supplementary material for: Incomplete lineage sorting and ancient admixture, and speciation without morphological change in ghost-worm cryptic species
Source: PeerJ. 2021 Feb 9;9:e10896. doi: 10.7717/peerj.10896 (PMC7879940; doi:10.7717/peerj.10896)
Supplement: Supplemental Information 1 — Sites where multiple species were found (i.e. sympatric sites) are given in bold. [file peerj-09-10896-s001.docx]

**Supplementary Table 1** Studied species, sites including GPS coordinates, population map IDs (as used in stacks) and number of specimens used in this study. Sites where multiple species were found (i.e. sympatric sites) are given in **bold**.

| **Species** | **Site** | **Latitude** | **Longitude** | **Population map** | **Number of specimens** |
| --- | --- | --- | --- | --- | --- |
| *Stygocapitella subterranea* | Ardtoe | 56.76923 | -5.88361 | Blue_ardtoe | **7** |
|  | Glenancross | 56.94472 | -5.85347 | Blue_glenancross | **4** |
|  | Ile Callot | 48.68713 | -3.92439 | Blue_ile | **3** |
|  | Keitum | 54.902 | 8.36766 | Blue_keitum | **6** |
|  | Little Gruinard | 57.85223 | -5.4533 | Blue_gruinard | **1** |
|  | **Hausstrand** | 55.01556 | 8.43736 | Blue_hausstrand | **1** |
|  | Nairn | 57.59653 | -3.84176 | Blue_nairn | **2** |
|  | Morsum | 54.87822 | 8.46527 | Blue_morsum | **3** |
|  | **Musselburough** | 55.94645 | -3.07624 | Blue_musselburough | **3** |
| *Stygocapitella josemariobrancoi* | Bristol Channel | 51.39973 | -3.19606 | Green_bristol | **3** |
|  | Ellenbogen | 55.04397 | 8.45172 | Green_ellenbogen | **5** |
|  | Gravesend | 51.44443 | 0.37764 | Green_gravesend | **4** |
|  | **Hausstrand** | 55.01556 | 8.43736 | Green_hausstrand | **4** |
|  | Hörnum | 54.75619 | 8.29466 | Green_hoernum | **4** |
|  | **Lubec** | 44.85482 | -66.98179 | Green_lubec | **2** |
|  | Plymouth | 50.34861 | -4.20071 | Green_plymouth | **3** |
|  | **Musselburough** | 55.94645 | -3.07624 | Green_musselburough | **6** |
|  | Saint Efflam | 48.684609 | -3.62247 | Green_St | **3** |
| *Stygocapitella westheidei* | Canoe Beach | 42.41962 | -70.90684 | Purple_canoe | **5** |
|  | **Lubec** | 44.85482 | -66.98179 | Purple_lubec | **5** |
|  | Reid State Park | 43.77628 | -69.73121 | Purple_reid | **5** |
|  | South Lubec | 44.82476 | -66.98917 | Purple_southlubec | **3** |
