# Supplementary material for: Incomplete lineage sorting and ancient admixture, and speciation without morphological change in ghost-worm cryptic species
Source: PeerJ. 2021 Feb 9;9:e10896. doi: 10.7717/peerj.10896 (PMC7879940; doi:10.7717/peerj.10896)
Supplement: Supplemental Information 2 — For each specimen we provide a sampling code, the collection site, a sampling code and the NCBI information for COI, 16S, 18S, ITS1. The column “Present in the final dataset” shows whether the specimen was removed due to ¿90% missing data, as shown in the final column. [file peerj-09-10896-s002.docx]

**Supplementary Table 2** Specimens used in this study. For each specimen we provide a sampling code, the collection site, a sampling code and the NCBI information for COI, 16S, 18S, ITS1. The column “Present in the final dataset” shows whether the specimen was removed due to >90% missing data, as shown in the final column.

| **Species** | **Site** | **Sampling**  **Code** | **COI** | **16S** | **18S** | **ITS1** | **Present in the final dataset?** | **% of missing data in the final dataset** |
| --- | --- | --- | --- | --- | --- | --- | --- | --- |
| *Stygocapitella subterranea* | Ardtoe | 320_02 | MN158516 | MN164320 |  |  | Yes | 58.2% |
|  | Ardtoe | 320_04 | MN158525 | MN164270 |  |  | Yes | 64.8% |
|  | Ardtoe | 320_06 | MN158526 | MN164298 | MN162926 |  | Yes | 63.7% |
|  | Ardtoe | 320_14 | MN158536 | MN164312 |  |  | Yes | 63.0% |
|  | Ardtoe | 320_15 | MN158523 | MN164299 |  |  | Yes | 62.7% |
|  | Ardtoe | 320_18 |  | XX000000 |  |  | Yes | 49.7% |
|  | Ardtoe | 320_20 |  | XX000000 |  |  | Removed | - |
|  | Ardtoe | 320_22 |  | XX000000 |  |  | Yes | 58.2% |
|  | Ardtoe | 320_24 |  | XX000000 |  |  | Removed | - |
|  | Ardtoe | 320_35 |  | XX000000 |  |  | Removed | - |
|  | Glenancross | 321_01 |  | XX000000 |  |  | Removed | - |
|  | Glenancross | 321_02 | MN158539 | MN164301 | MN162952 |  | Yes | 52.7% |
|  | Glenancross | 321_03 | MN158542 | MN164303 | MN162940 |  | Yes | 61.9% |
|  | Glenancross | 321_04 | MN158534 | MN164304 | MN162941 |  | Yes | 34.3% |
|  | Glenancross | 321_05 | MN158530 | MN164305 |  |  | Yes | 27.4% |
|  | Hausstrand | 169_57 | MN158571 | MN164337 |  |  | Yes | 67.0% |
|  | Île Callot | 403_03 | MN158508 | MN164265 | MN162950 | MN162762 | Yes | 44.6% |
|  | Île Callot | 403_04 | MN158567 | MN164266 | MN162942 |  | Yes | 36.3% |
|  | Île Callot | 403_05 | MN158509 | MN164267 | MN162930 |  | Yes | 51.4% |
|  | Île Callot | 403_07 | MN158545 | MN164322 |  |  | Removed | - |
|  | Île Callot | 403_08 | MN158554 | MN164273 |  |  | Removed | - |
|  | Keitum | 398_04 | MN158510 | MN164275 | MN162956 |  | Yes | 65.4% |
|  | Keitum | 398_05 | MN158546 | MN164276 |  |  | Yes | 66.6% |
|  | Keitum | 398_06 | MN158550 | MN164277 |  |  | Yes | 79.1% |
|  | Keitum | 398_07 | MN158511 | MN164278 |  |  | Yes | 79.3% |
|  | Keitum | 398_08 | MN158512 | MN164297 |  |  | Yes | 78.8% |
|  | Keitum | 398_09 | MN158544 | MN164279 |  |  | Yes | 80.1% |
|  | Keitum | 398_10 | MN158513 | MN164269 |  | MN162763 | Removed | - |
|  | Little Gruinard | 322_01 | MN158576 | MN164314 |  |  | Removed | - |
|  | Little Gruinard | 322_02 | MN158577 | MN164335 |  |  | Yes | 16.9% |
|  | Morsum | 227_01 | MN158519 | MN164285 | MN162935 |  | Yes | 24.9% |
|  | Morsum | 227_03 | MN158573 | MN164271 | MN162955 |  | Yes | 33.9% |
|  | Morsum | 227_04 | MN158521 | MN164272 | MN162936 |  | Removed | - |
|  | Morsum | 227_06 | MN158527 | MN164294 |  |  | Removed | - |
|  | Morsum | 227_09 |  | MN164296 |  |  | Yes | 40.0% |
|  | Morsum | 296_21 |  | XX000000 |  |  | Removed | - |
|  | Musselburgh | 324_03 | MN158565 | MN164331 |  |  | Yes | 30.9% |
|  | Musselburgh | 324_05 | MN158549 | MN164332 |  | MN162764 | Yes | 28.3% |
|  | Musselburgh | 324_29 |  | XX000000 |  |  | Removed | - |
|  | Musselburgh | 324_30 |  | XX000000 |  |  | Removed | - |
|  | Musselburgh | 324_32 |  | XX000000 |  |  | Removed | - |
|  | Musselburgh | 324_33 |  | XX000000 |  |  | Yes | 63.7% |
|  | Musselburgh | 324_44 |  | XX000000 |  |  | Removed | - |
|  | Nairn | 323_01 | MN158531 | MN164329 |  |  | Yes | 20.3% |
|  | Nairn | 323_02 | MN158533 | MN164333 |  |  | Yes | 22.1% |
|  | Nairn | 323_03 | MN158552 | MN164330 |  |  | Removed | - |
|  | Nairn | 323_04 | MN158532 | MN164334 | MN162957 | MN162765 | Removed | - |
| *Stygocapitella josemariobrancoi* | Bristol Channel | 422_01 | MN158387 | MN164135 | MN162970 | MN162799 | Removed | - |
|  | Bristol Channel | 422_02 | MN158399 | MN164136 | MN162971 | MN162803 | Removed | - |
|  | Bristol Channel | 422_03 | MN158400 | MN164144 | MN162972 | MN162805 | Removed | - |
|  | Bristol Channel | 422_04 | MN158388 | MN164148 | MN162978 | MN162808 | Yes | 81.7% |
|  | Bristol Channel | 422_05 | MN158413 | MN164176 |  |  | Yes | 79.0% |
|  | Ellenbogen | 222_01 | MN158440 | MN164199 | MN162982 | MN162809 | Yes | 87.1% |
|  | Ellenbogen | 222_02 | MN158418 | MN164153 | MN162975 | MN162810 | Yes | 88.7% |
|  | Ellenbogen | 222_03 | MN158396 | MN164171 | MN162979 | MN162832 | Yes | 88.2% |
|  | Ellenbogen | 222_04 | MN158416 | MN164142 | MN162984 | MN162811 | Yes | 86.7% |
|  | Ellenbogen | 222_13 |  | MN164143 |  |  | Removed | - |
|  | Gravesend | 424_01 | MN158447 | MN164201 |  | MN162842 | Removed | - |
|  | Gravesend | 424_02 | MN158436 | MN164151 |  |  | Removed | - |
|  | Gravesend | 424_03 | MN158463 | MN164211 |  |  | Removed | - |
|  | Gravesend | 424_04 | MN158448 | MN164202 |  |  | Removed | - |
|  | Gravesend | 424_05 | MN158449 | MN164213 |  | MN162843 | Yes | 90.1% |
|  | Gravesend | 424_06 | MN158450 | MN164203 |  | MN162846 | Removed | - |
|  | Gravesend | 424_07 | MN158451 | MN164212 |  | MN162841 | Removed | - |
|  | Hörnum | 169_06 | MN158391 | MN164180 |  | MN162828 | Yes | 87.5% |
|  | Hörnum | 169_07 | MN158404 | MN164192 |  | MN162822 | Yes | 88.7% |
|  | Hörnum | 169_08 | MN158439 | MN164159 |  |  | Removed | - |
|  | Hörnum | 169_09 | MN158424 | MN164165 | MN162973 | MN162839 | Yes | 90.8% |
|  | Hörnum | 1 | MN158392 | MN164174 | MN162974 | MN162825 | Removed | - |
|  | Hausstrand | 169_58 | MN158417 | MN164164 | MN162976 | MN162813 | Yes | 90.1% |
|  | Hausstrand | 219_05 | MN158442 | MN164170 |  | MN162818 | Yes | 88.0% |
|  | Hausstrand | 219_06 | MN158443 | MN164163 |  | MN162814 | Yes | 87.0% |
|  | Hausstrand | 219_11 | MN158427 |  |  |  | Removed | - |
|  | Hausstrand | 219_12 |  | MN164162 |  |  | Yes | 92.1% |
|  | Lubec | 429_01 | MN158444 | MN164138 | MN162963 | MN162850 | Removed | - |
|  | Lubec | 429_08 | MN158429 | MN164185 | MN162967 | MN162851 | Yes | 85.0% |
|  | Plymouth | 421_01 | MN158471 | MN164224 | MN162964 | MN162852 | Removed | - |
|  | Plymouth | 421_02 | MN158474 | MN164225 |  |  | Removed | - |
|  | Plymouth | 421_03 | MN158473 | MN164228 |  | MN162865 | Removed | - |
|  | Plymouth | 421_04 | MN158438 | MN164186 |  |  | Removed | - |
|  | Plymouth | 421_05 | MN158475 | MN164229 |  | MN162853 | Removed | - |
|  | Musselburgh | 324_02 | MN158457 | MN164215 |  |  | Yes | 90.1% |
|  | Musselburgh | 324_07 | MN158458 | MN164217 |  |  | Yes | 89.7% |
|  | Musselburgh | 324_09 | MN158460 | MN164219 |  |  | Yes | 90.1% |
|  | Musselburgh | 324_23 |  | XX00000 |  |  | Removed | - |
|  | Musselburgh | 324_25 |  | XX00000 |  |  | Removed | - |
|  | Musselburgh | 324_34 |  | XX00000 |  |  | Removed | - |
|  | Musselburgh | 324_36 |  | XX00000 |  |  | Removed | - |
|  | Musselburgh | 324_42 |  | XX00000 |  |  | Removed | - |
|  | Musselburgh | 324_50 |  | XX00000 |  |  | Removed | - |
|  | Musselburgh | 324_52 |  | XX00000 |  |  | Removed | - |
|  | St. Efflam | 401_03 | MN158454 | MN164214 | MN162965 |  | Yes | 71.6% |
|  | St. Efflam | 401_04 | MN158437 | MN164194 |  | MN162857 | Yes | 68.8% |
|  | St. Efflam | 401_05 | MN158434 | MN164146 |  |  | Yes | 77.2% |
|  | St. Efflam | 401_06 | MN158445 | MN164145 |  | MN162859 | Removed | - |
|  | St. Efflam | 401_07 | MN158469 | MN164210 |  | MN162866 | Removed | - |
| *Stygocapitella westheidei* | Canoe Beach | 426_01 | MN158481 | MN164233 | MN162928 | MN162768 | Yes | 52.4% |
|  | Canoe Beach | 426_02 | MN158503 |  | MN162960 | MN162770 | Removed | - |
|  | Canoe Beach | 426_03 | MN158504 | MN164234 | MN162939 | MN162794 | Yes | 43.3% |
|  | Canoe Beach | 426_04 | MN158502 | MN164235 | MN162948 | MN162784 | Yes | 43.4% |
|  | Canoe Beach | 426_05 | MN158507 | MN164236 |  |  | Yes | 42.0% |
|  | Canoe Beach | 426_06 | MN158486 | MN164237 |  | MN162795 | Yes | 67.1% |
|  | Canoe Beach | 426_07 | MN158501 | MN164254 |  | MN162771 | Removed | - |
|  | Lubec | 429_02 | MN158482 |  | MN162934 | MN162775 | Yes | 68.1% |
|  | Lubec | 429_03 |  | MN164255 |  | MN162776 | Removed | - |
|  | Lubec | 429_04 |  | MN164240 |  |  | Yes | 75.0% |
|  | Lubec | 429_05 |  | MN164256 |  | MN162777 | Yes | 82.2% |
|  | Lubec | 429_06 |  | MN164251 |  | MN162778 | Removed | - |
|  | Lubec | 429_07 |  |  |  | MN162793 | Yes | 81.9% |
|  | Lubec | 429_09 | MN158490 | MN164257 |  | MN162779 | Yes | 64.4% |
|  | Reid State Park | 427_01 | MN158484 | MN164259 | MN162943 | MN162781 | Removed | - |
|  | Reid State Park | 427_02 | MN158495 | MN164260 | MN162931 | MN162772 | Removed | - |
|  | Reid State Park | 427_03 | MN158487 | MN164243 | MN162937 | MN162773 | Yes | 43.1% |
|  | Reid State Park | 427_04 | MN158485 | MN164252 | MN162944 | MN162786 | Yes | 38.9% |
|  | Reid State Park | 427_05 | MN158499 | MN164244 |  |  | Yes | 47.8% |
|  | Reid State Park | 427_06 | MN158500 | MN164245 |  | MN162787 | Yes | 50.3% |
|  | Reid State Park | 427_07 | MN158492 | MN164261 |  | MN162789 | Yes | 69.2% |
|  | South Lubec | 428_01 | MN158491 | MN164263 | MN162946 | MN162791 | Yes | 67.8% |
|  | South Lubec | 428_02 | MN158498 | MN164247 | MN162969 | MN162790 | Yes | 67.2% |
|  | South Lubec | 428_03 | MN158488 | MN164248 | MN162947 | MN162769 | Yes | 71.2% |
