# Supplementary figures and images for: Incomplete lineage sorting and ancient admixture, and speciation without morphological change in ghost-worm cryptic species
Source: PeerJ. 2021 Feb 9;9:e10896. doi: 10.7717/peerj.10896 (PMC7879940; doi:10.7717/peerj.10896)

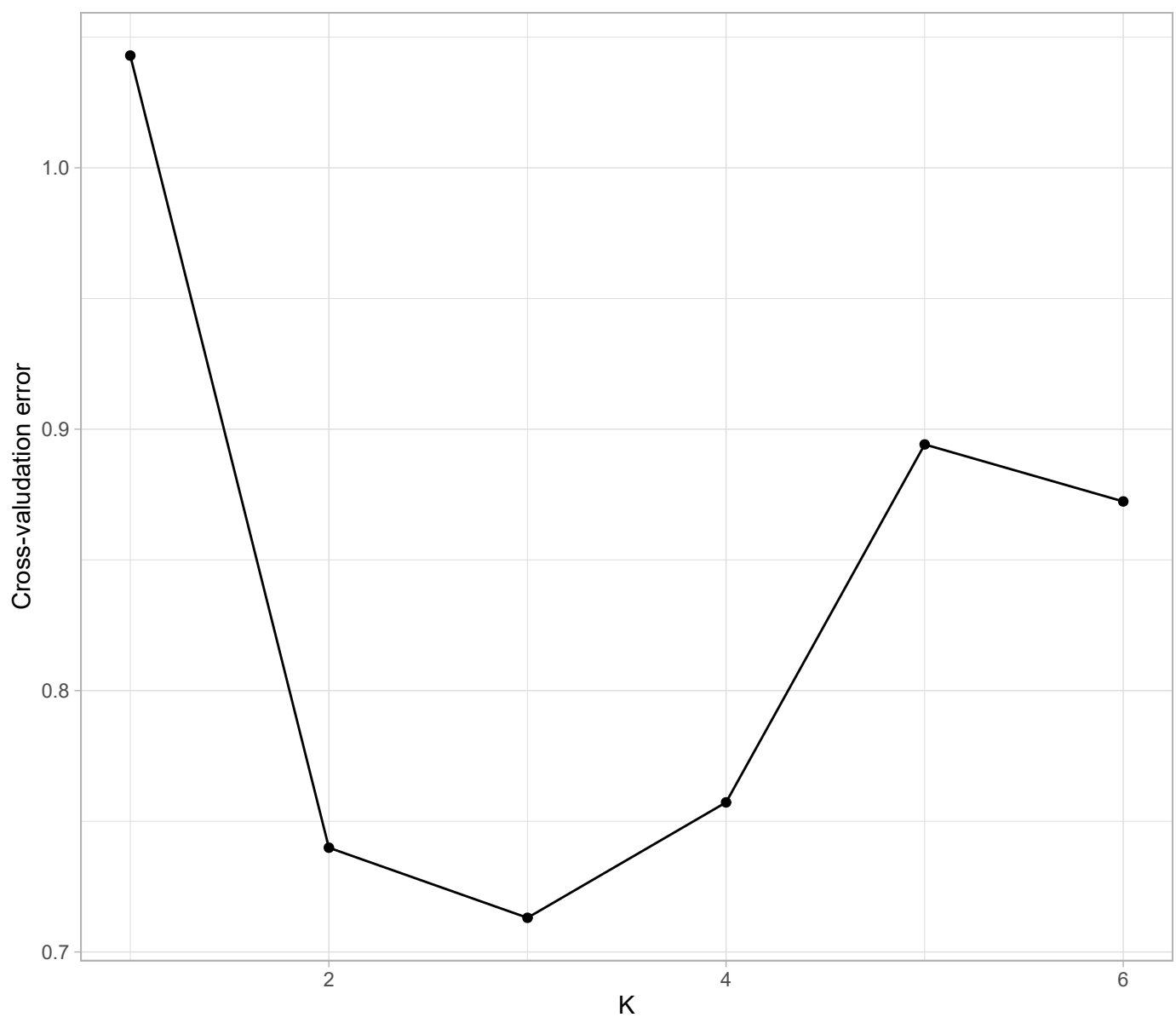

Supplement: Figure S1 — The cross-validation error is provided in the Y axis and the different K in the X axis. [file peerj-09-10896-s003.pdf]

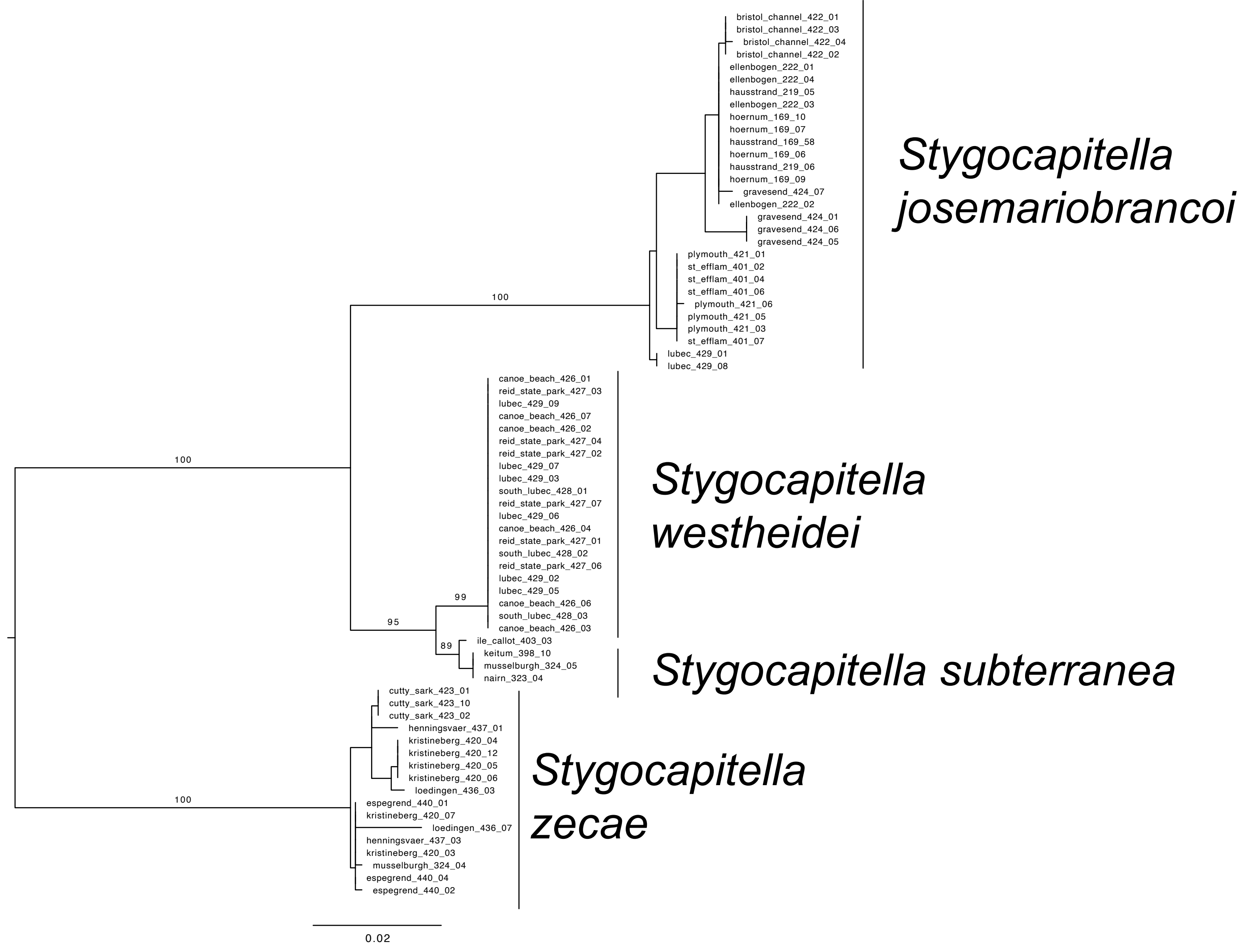

Supplement: Figure S3 — Bootstrap support for the four species is provided above the branches. Stygocapitella zecae is added as outgroup. [file peerj-09-10896-s005.pdf]

Species

*S. subterranea*

*S. josemariobrancoi*

*S. westheidei*

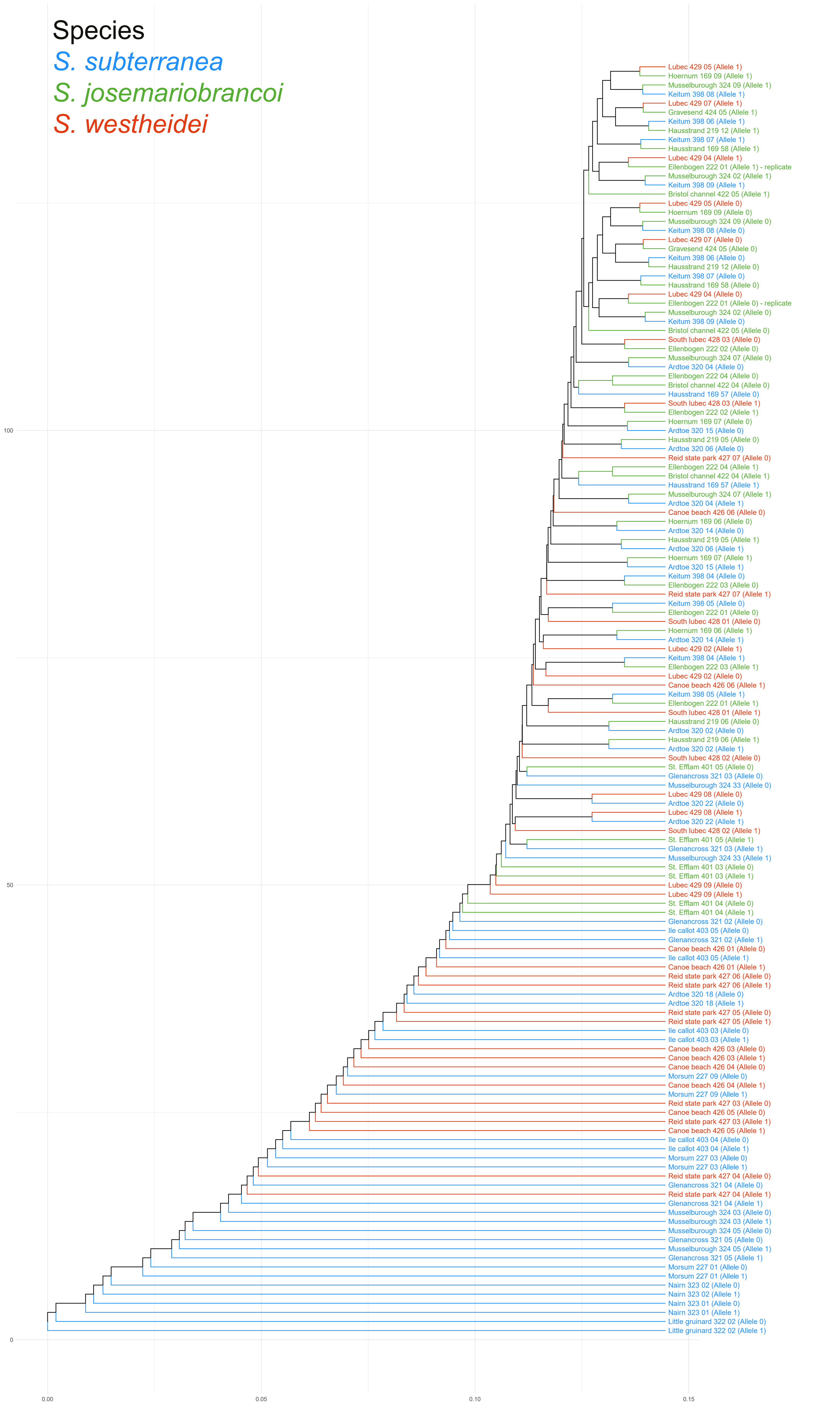

Supplement: Figrue S6 — Specimens from different species are coloured differently. [file peerj-09-10896-s008.pdf]

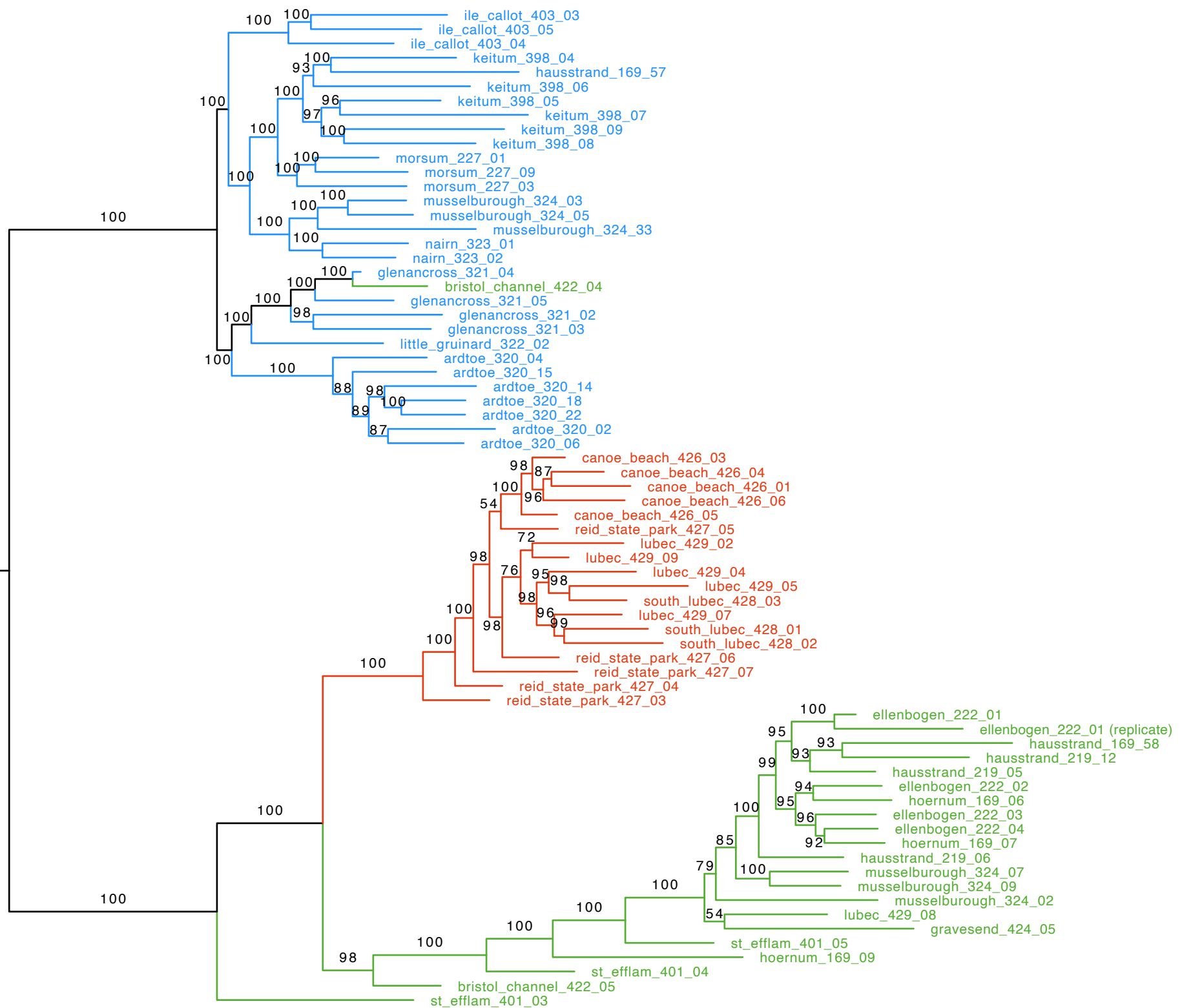

0.003

Supplement: Figure S7 — Rad-seq alleles (0 and 1) were converted into a consensus sequence. Bootstrap support is provided for the main branches. Coloration follows species with blue representing Stygocapitella subterranea, green representing S. josemariobrancoi, and orange S. westheidei. [file peerj-09-10896-s009.pdf]
