# Supplementary material for: Incomplete lineage sorting and ancient admixture, and speciation without morphological change in ghost-worm cryptic species
Source: PeerJ. 2021 Feb 9;9:e10896. doi: 10.7717/peerj.10896 (PMC7879940; doi:10.7717/peerj.10896)
Supplement: Figure S2 — Bootstrap support for the four species is provided above the branches. Stygocapitella zecae is added as outgroup. [file peerj-09-10896-s004.pdf]

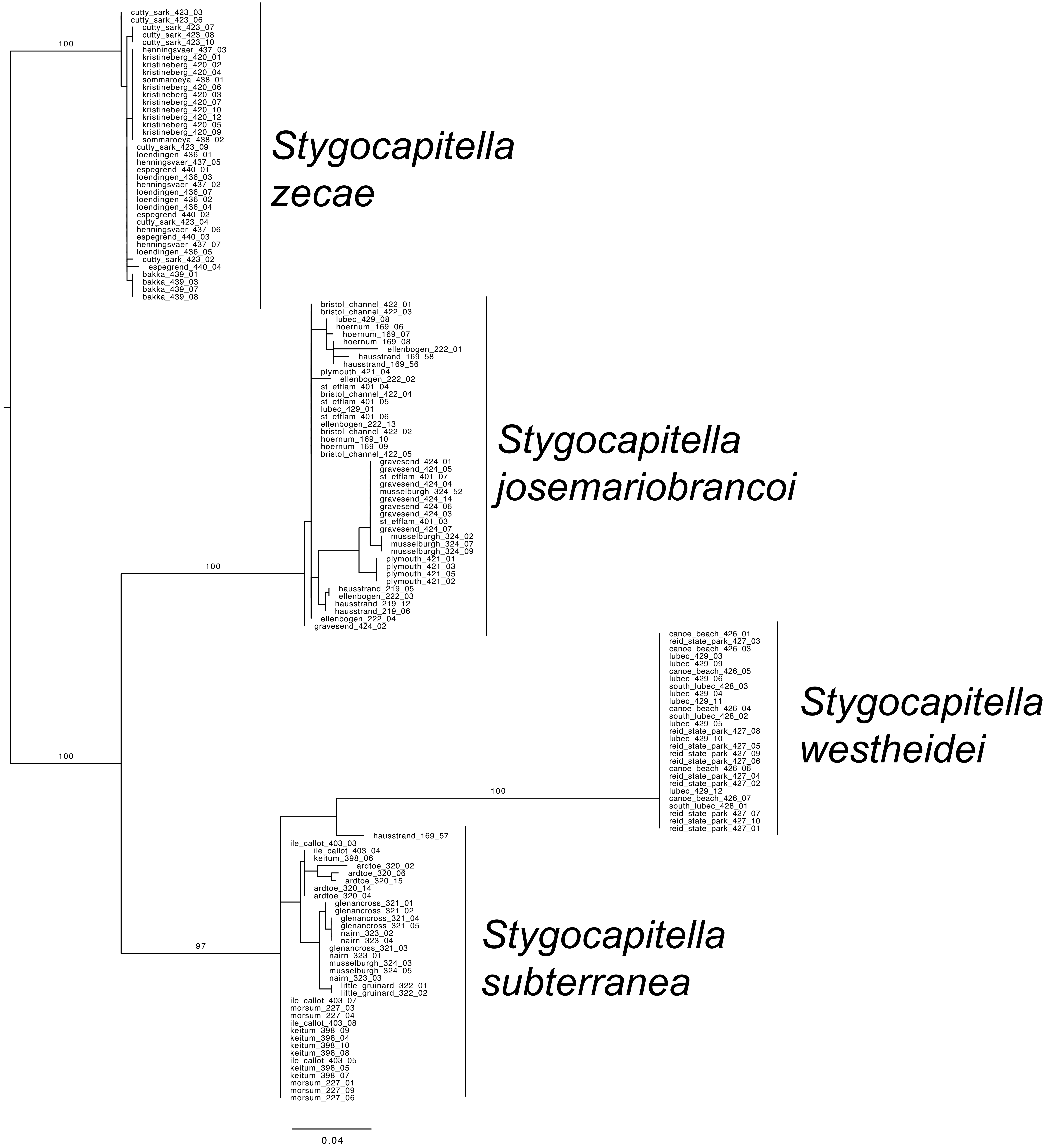

cutty_sark_423_03
cutty_sark_423_06
cutty_sark_423_07
cutty_sark_423_08
cutty_sark_423_10
henningsvaer_437_03
kristineberg_420_01
kristineberg_420_02
kristineberg_420_04
sommaroeya_438_01
kristineberg_420_06
kristineberg_420_03
kristineberg_420_07
kristineberg_420_10
kristineberg_420_12
kristineberg_420_05
kristineberg_420_09
sommaroeya_438_02
cutty_sark_423_09
loendingen_436_01
henningsvaer_437_05
espegrend_440_01
loendingen_436_03
henningsvaer_437_02
loendingen_436_07
loendingen_436_02
loendingen_436_04
espegrend_440_02
cutty_sark_423_04
henningsvaer_437_06
espegrend_440_03
henningsvaer_437_07
loendingen_436_05
cutty_sark_423_02
espegrend_440_04
bakka_439_01
bakka_439_03
bakka_439_07
bakka_439_08
100
Stygocapitella zecae
bristol_channel_422_01
bristol_channel_422_03
lubec_429_08
hoernum_169_06
hoernum_169_07
hoernum_169_08
ellenbogen_222_01
hausstrand_169_58
hausstrand_169_56
plymouth_421_04
ellenbogen_222_02
st_efflam_401_04
bristol_channel_422_04
st_efflam_401_05
lubec_429_01
st_efflam_401_06
ellenbogen_222_13
bristol_channel_422_02
hoernum_169_10
hoernum_169_09
bristol_channel_422_05
gravesend_424_01
gravesend_424_05
st_efflam_401_07
gravesend_424_04
musselburgh_324_52
gravesend_424_14
gravesend_424_06
gravesend_424_03
st_efflam_401_03
gravesend_424_07
musselburgh_324_02
musselburgh_324_07
musselburgh_324_09
plymouth_421_01
plymouth_421_03
plymouth_421_05
plymouth_421_02
hausstrand_219_05
ellenbogen_222_03
hausstrand_219_12
hausstrand_219_06
ellenbogen_222_04
gravesend_424_02
100
Stygocapitella josemariobrancoi
canoe_beach_426_01
reid_state_park_427_03
canoe_beach_426_03
lubec_429_03
lubec_429_09
canoe_beach_426_05
lubec_429_06
south_lubec_428_03
lubec_429_04
lubec_429_11
canoe_beach_426_04
south_lubec_428_02
lubec_429_05
reid_state_park_427_08
lubec_429_10
reid_state_park_427_05
reid_state_park_427_09
reid_state_park_427_06
canoe_beach_426_06
reid_state_park_427_04
reid_state_park_427_02
lubec_429_12
canoe_beach_426_07
south_lubec_428_01
reid_state_park_427_07
reid_state_park_427_10
reid_state_park_427_01
100
Stygocapitella westheidei
100
hausstrand_169_57
ile_callot_403_03
ile_callot_403_04
keitum_398_06
ardtoe_320_02
ardtoe_320_06
ardtoe_320_15
ardtoe_320_14
ardtoe_320_04
glenancross_321_01
glenancross_321_02
glenancross_321_04
glenancross_321_05
nairn_323_02
nairn_323_04
glenancross_321_03
nairn_323_01
musselburgh_324_03
musselburgh_324_05
nairn_323_03
little_gruinard_322_01
little_gruinard_322_02
ile_callot_403_07
morsum_227_03
morsum_227_04
ile_callot_403_08
keitum_398_09
keitum_398_04
keitum_398_10
keitum_398_08
ile_callot_403_05
keitum_398_05
keitum_398_07
morsum_227_01
morsum_227_09
morsum_227_06
97
Stygocapitella subterranea
0.04
