# Supplementary material for: Incomplete lineage sorting and ancient admixture, and speciation without morphological change in ghost-worm cryptic species
Source: PeerJ. 2021 Feb 9;9:e10896. doi: 10.7717/peerj.10896 (PMC7879940; doi:10.7717/peerj.10896)
Supplement: Figure S4 — Bootstrap support for the four species is provided above the branches. Stygocapitella zecae is added as outgroup. [file peerj-09-10896-s006.pdf]

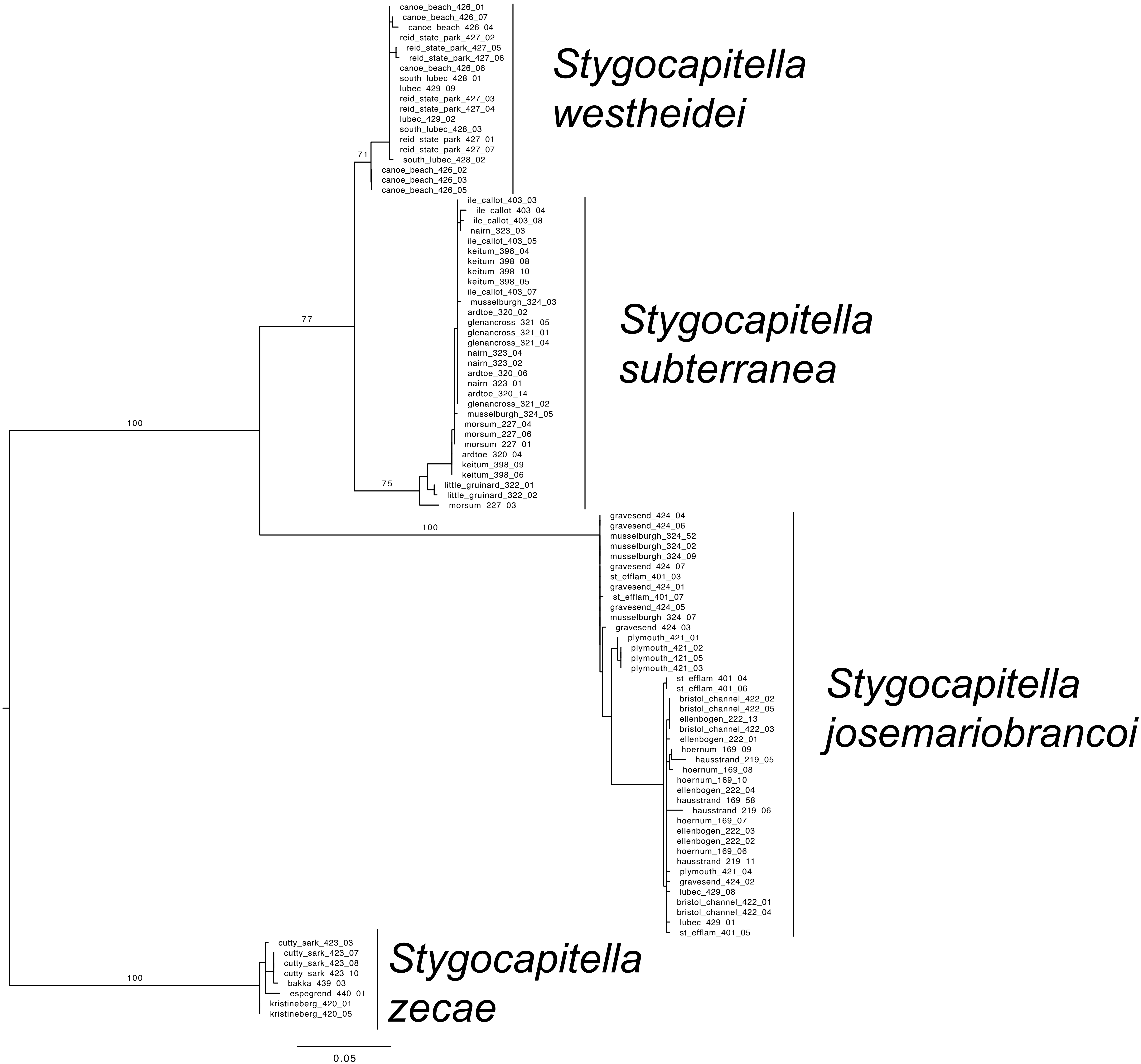
Stygocapitella westheidei
canoe_beach_426_01
canoe_beach_426_07
canoe_beach_426_04
reid_state_park_427_02
reid_state_park_427_05
reid_state_park_427_06
canoe_beach_426_06
south_lubec_428_01
lubec_429_09
reid_state_park_427_03
reid_state_park_427_04
lubec_429_02
south_lubec_428_03
reid_state_park_427_01
reid_state_park_427_07
south_lubec_428_02
canoe_beach_426_02
canoe_beach_426_03
canoe_beach_426_05
71
77
100
Stygocapitella subterranea
ile_callot_403_03
ile_callot_403_04
ile_callot_403_08
nairn_323_03
ile_callot_403_05
keitum_398_04
keitum_398_08
keitum_398_10
keitum_398_05
ile_callot_403_07
musselburgh_324_03
ardtoe_320_02
glenancross_321_05
glenancross_321_01
glenancross_321_04
nairn_323_04
nairn_323_02
ardtoe_320_06
nairn_323_01
ardtoe_320_14
glenancross_321_02
musselburgh_324_05
morsum_227_04
morsum_227_06
morsum_227_01
ardtoe_320_04
keitum_398_09
keitum_398_06
75
little_gruinard_322_01
little_gruinard_322_02
morsum_227_03
100
Stygocapitella josemariobrancoi
gravesend_424_04
gravesend_424_06
musselburgh_324_52
musselburgh_324_02
musselburgh_324_09
gravesend_424_07
st_efflam_401_03
gravesend_424_01
st_efflam_401_07
gravesend_424_05
musselburgh_324_07
gravesend_424_03
plymouth_421_01
plymouth_421_02
plymouth_421_05
plymouth_421_03
st_efflam_401_04
st_efflam_401_06
bristol_channel_422_02
bristol_channel_422_05
ellenbogen_222_13
bristol_channel_422_03
ellenbogen_222_01
hoernum_169_09
hausstrand_219_05
hoernum_169_08
hoernum_169_10
ellenbogen_222_04
hausstrand_169_58
hausstrand_219_06
hoernum_169_07
ellenbogen_222_03
ellenbogen_222_02
hoernum_169_06
hausstrand_219_11
plymouth_421_04
gravesend_424_02
lubec_429_08
bristol_channel_422_01
bristol_channel_422_04
lubec_429_01
st_efflam_401_05
100
Stygocapitella zecae
cutty_sark_423_03
cutty_sark_423_07
cutty_sark_423_08
cutty_sark_423_10
bakka_439_03
espegrend_440_01
kristineberg_420_01
kristineberg_420_05
0.05

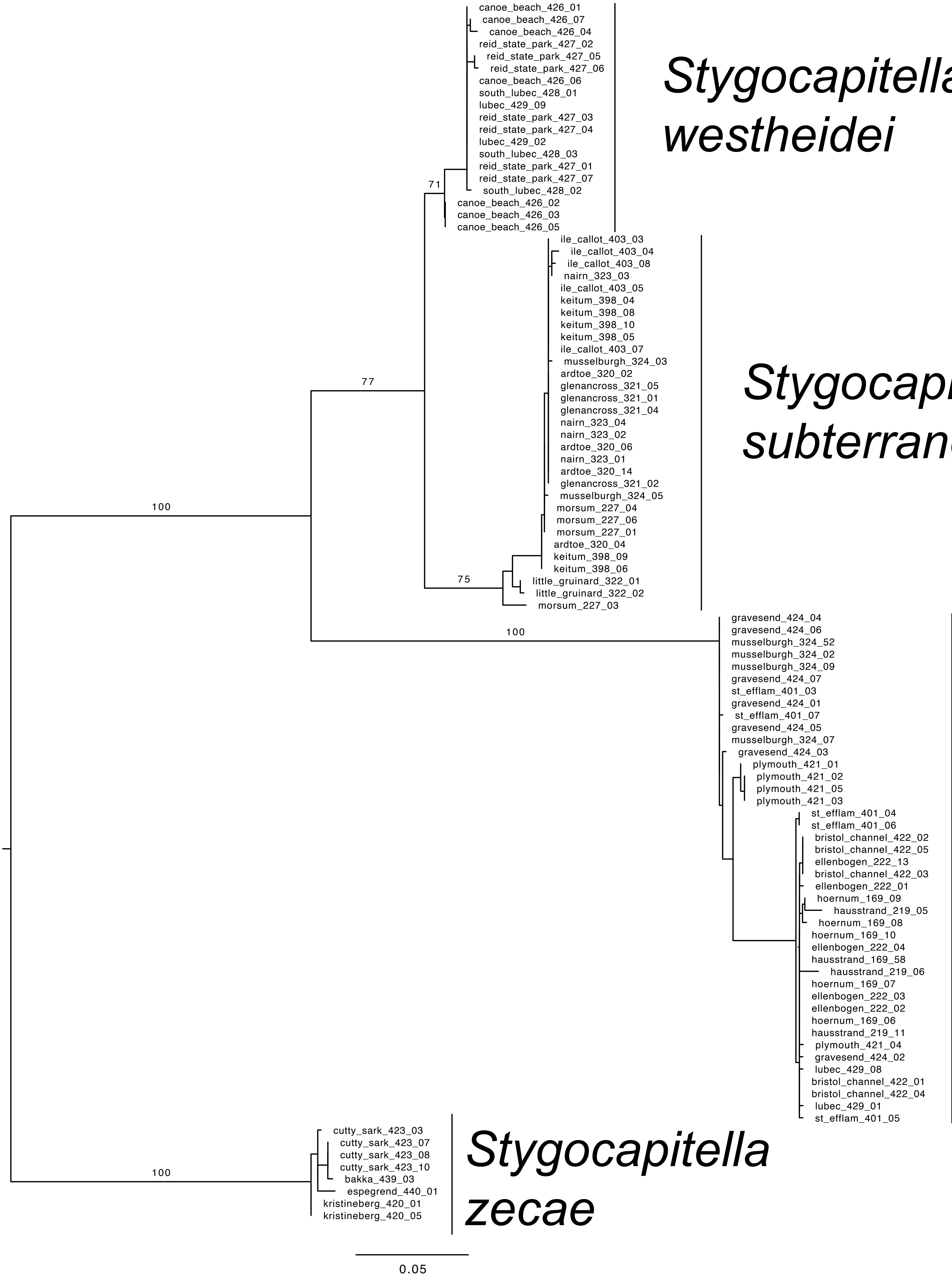
canoe_beach_426_01
canoe_beach_426_07
canoe_beach_426_04
reid_state_park_427_02
reid_state_park_427_05
reid_state_park_427_06
canoe_beach_426_06
south_lubec_428_01
lubec_429_09
reid_state_park_427_03
reid_state_park_427_04
lubec_429_02
south_lubec_428_03
reid_state_park_427_01
reid_state_park_427_07
south_lubec_428_02
canoe_beach_426_02
canoe_beach_426_03
canoe_beach_426_05
71
Stygocapitell
westheidei
ile_callot_403_03
ile_callot_403_04
ile_callot_403_08
nairn_323_03
ile_callot_403_05
keitum_398_04
keitum_398_08
keitum_398_10
keitum_398_05
ile_callot_403_07
musselburgh_324_03
ardtoe_320_02
glenancross_321_05
glenancross_321_01
glenancross_321_04
nairn_323_04
nairn_323_02
ardtoe_320_06
nairn_323_01
ardtoe_320_14
glenancross_321_02
musselburgh_324_05
morsum_227_04
morsum_227_06
morsum_227_01
ardtoe_320_04
keitum_398_09
keitum_398_06
little_gruinard_322_01
little_gruinard_322_02
morsum_227_03
77
75
100
Stygocapi
subterran
gravesend_424_04
gravesend_424_06
musselburgh_324_52
musselburgh_324_02
musselburgh_324_09
gravesend_424_07
st_efflam_401_03
gravesend_424_01
st_efflam_401_07
gravesend_424_05
musselburgh_324_07
gravesend_424_03
plymouth_421_01
plymouth_421_02
plymouth_421_05
plymouth_421_03
st_efflam_401_04
st_efflam_401_06
bristol_channel_422_02
bristol_channel_422_05
ellenbogen_222_13
bristol_channel_422_03
ellenbogen_222_01
hoernum_169_09
hausstrand_219_05
hoernum_169_08
hoernum_169_10
ellenbogen_222_04
hausstrand_169_58
hausstrand_219_06
hoernum_169_07
ellenbogen_222_03
ellenbogen_222_02
hoernum_169_06
hausstrand_219_11
plymouth_421_04
gravesend_424_02
lubec_429_08
bristol_channel_422_01
bristol_channel_422_04
lubec_429_01
st_efflam_401_05
100
cutty_sark_423_03
cutty_sark_423_07
cutty_sark_423_08
cutty_sark_423_10
bakka_439_03
espegrend_440_01
kristineberg_420_01
kristineberg_420_05
100
Stygocapitella
zecae
0.05
