# Supplementary material for: Incomplete lineage sorting and ancient admixture, and speciation without morphological change in ghost-worm cryptic species
Source: PeerJ. 2021 Feb 9;9:e10896. doi: 10.7717/peerj.10896 (PMC7879940; doi:10.7717/peerj.10896)
Supplement: Figure S5 — Bootstrap support for the four species is provided above the branches. Stygocapitella zecae is added as outgroup. [file peerj-09-10896-s007.pdf]

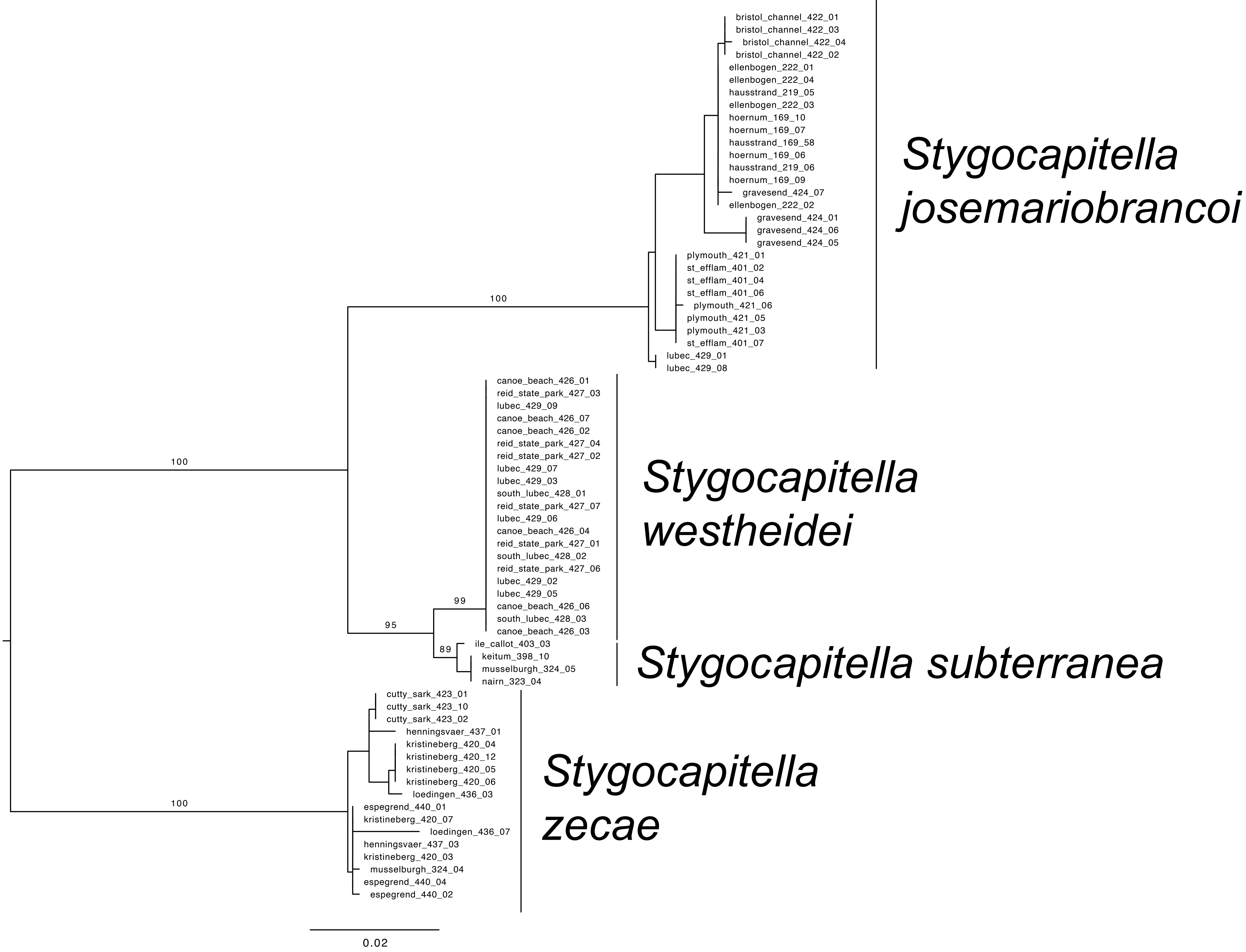

bristol_channel_422_01
bristol_channel_422_03
bristol_channel_422_04
bristol_channel_422_02
ellenbogen_222_01
ellenbogen_222_04
hausstrand_219_05
ellenbogen_222_03
hoernum_169_10
hoernum_169_07
hausstrand_169_58
hoernum_169_06
hausstrand_219_06
hoernum_169_09
gravesend_424_07
ellenbogen_222_02
gravesend_424_01
gravesend_424_06
gravesend_424_05
plymouth_421_01
st_efflam_401_02
st_efflam_401_04
st_efflam_401_06
plymouth_421_06
plymouth_421_05
plymouth_421_03
st_efflam_401_07
lubec_429_01
lubec_429_08
Stygocapitella josemariobrancoi
100
100
canoe_beach_426_01
reid_state_park_427_03
lubec_429_09
canoe_beach_426_07
canoe_beach_426_02
reid_state_park_427_04
reid_state_park_427_02
lubec_429_07
lubec_429_03
south_lubec_428_01
reid_state_park_427_07
lubec_429_06
canoe_beach_426_04
reid_state_park_427_01
south_lubec_428_02
reid_state_park_427_06
lubec_429_02
lubec_429_05
canoe_beach_426_06
south_lubec_428_03
canoe_beach_426_03
Stygocapitella westheidei
99
95
89
ile_callot_403_03
keitum_398_10
musselburgh_324_05
nairn_323_04
Stygocapitella subterranea
cutty_sark_423_01
cutty_sark_423_10
cutty_sark_423_02
henningsvaer_437_01
kristineberg_420_04
kristineberg_420_12
kristineberg_420_05
kristineberg_420_06
loedingen_436_03
espegrend_440_01
kristineberg_420_07
loedingen_436_07
henningsvaer_437_03
kristineberg_420_03
musselburgh_324_04
espegrend_440_04
espegrend_440_02
Stygocapitella zecae
100
0.02
