# Supplementary material for: Incomplete lineage sorting and ancient admixture, and speciation without morphological change in ghost-worm cryptic species
Source: PeerJ. 2021 Feb 9;9:e10896. doi: 10.7717/peerj.10896 (PMC7879940; doi:10.7717/peerj.10896)
Supplement: Figure S8 — Different models are depicted in the X axis and have different colours, AIC values are given in the Y axis. Species names are reduced with ‘s’ representing Stygocapitella subterranea, ‘i’ representing Stygocapitella josemariobrancoi, and ‘w’ representing Stygocapitella westheidei. [file peerj-09-10896-s010.pdf]

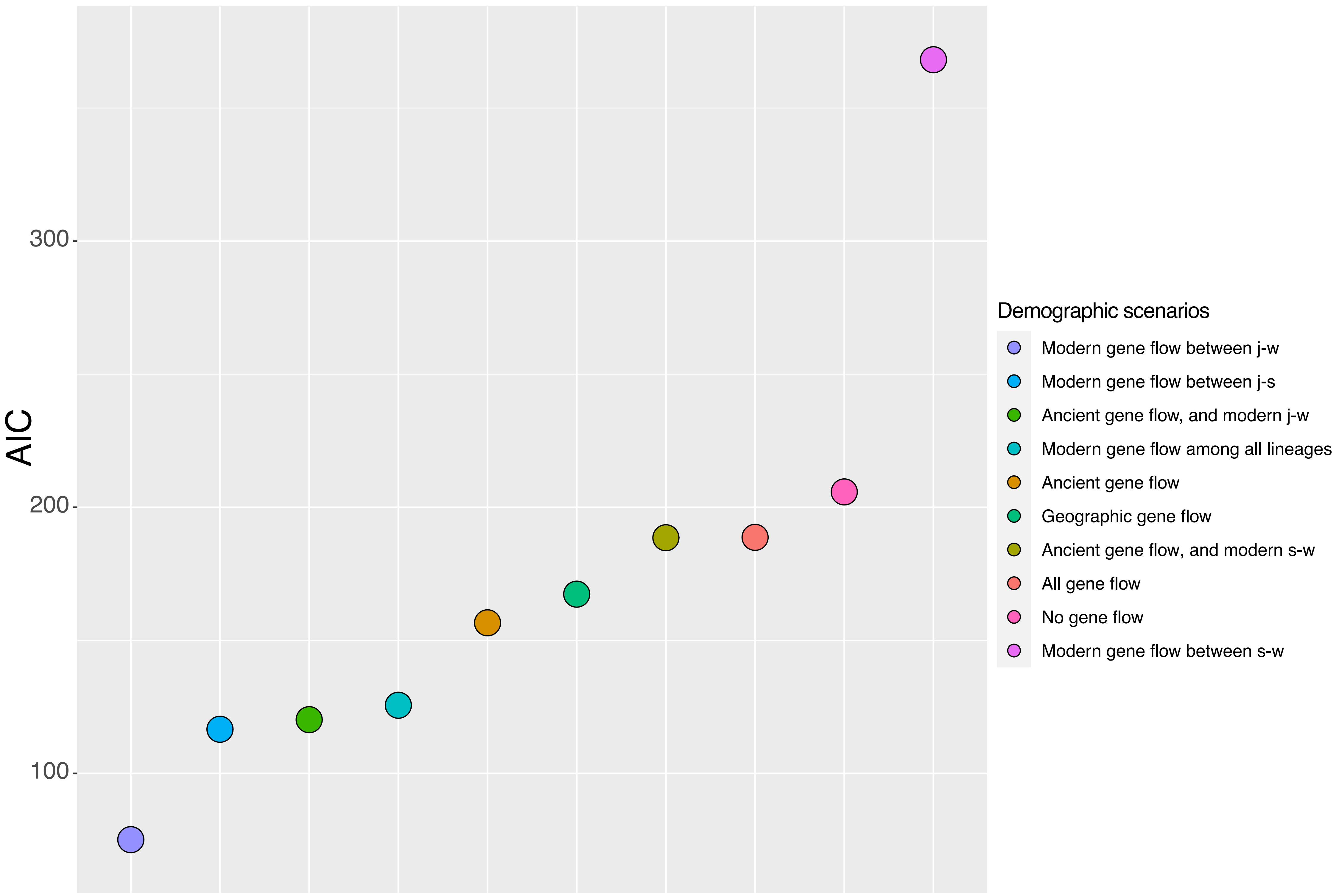

AIC
300
200
100
Demographic scenarios
Modern gene flow between j-w
Modern gene flow between j-s
Ancient gene flow, and modern j-w
Modern gene flow among all lineages
Ancient gene flow
Geographic gene flow
Ancient gene flow, and modern s-w
All gene flow
No gene flow
Modern gene flow between s-w
